# *Taenia taeniaeformis* in Rat Favors Protracted Skin Lesions Caused by *Sporothrix schenckii* Infection: Dectin-1 and IL-17 Are Dispensable for Clearance of This Fungus

**DOI:** 10.1371/journal.pone.0052514

**Published:** 2012-12-20

**Authors:** Xiaohui Zhang, Jing Zhang, Huaiqiu Huang, Ruzeng Xue, Xuchu Hu, Meirong Li, Yi Zhong, Liyan Yuan

**Affiliations:** 1 Department of Dermatology and Venereology, Third Affiliated Hospital of Sun Yat-sen University, Guangzhou, Guangdong Province, China; 2 Department of Dermatology, Guangdong Provincial Center for Skin Diseases and STIs Control and Prevention, Guangzhou, Guangdong Province, China; 3 Department of Dermatology and Venereology, Sun Yat-sen Memorial Hospital, Sun Yat-sen University, Guangzhou, Guangdong Province, China; 4 Department of Parasitology, Zhongshan School of Medicine, Sun Yat-sen University, Guangzhou, Guangdong Province, China; University of Alabama at Birmingham, United States of America

## Abstract

We occasionally found that cestode *Taenia taeniaeformis* in rats favored *Sporothrix schenckii* infection and survival, causing protracted cutaneous lesions. In this study, we compared the pathology and cytokines profile of rats co-infected with the two pathogens and infected with *S. schenckii* alone to explore underlying mechanisms. In the co-infection group, there was high expression of β-glucan receptor Dectin-1 in the cutaneous lesions and no multinucleated giant cells, but in the *S. schenckii* infection group the opposite was observed. Cytokines profiles demonstrated an expected finding that IL-4, commonly expressed in helminth and fungus infection, is undetectable in the two infection groups. In the single fungal infection group, cytokines IFN-γ, IL-10 and IL-17 kept increasing in the first few weeks of infection to a peak which was followed by gradual decrease. This study showed that Dectin-1 and IL-17, which were believed to be the major anti-fungus mechanisms, are Th2 independent and dispensable for clearance of *S. schenckii* infection, suggesting that *S. schenckii* has a different molecular recognition pattern and evokes anti-infection mechanisms other than Dectin-1 and IL-17.

## Introduction

Sporotrichosis is a sub-acute or chronic infection caused by the dimorphic fungus *Sporothrix schenckii*. It occurs worldwide and is prevalent in tropical and subtropical regions [1], [2], such as China, Japan, Latin America, and South Africa. *S. schenckii* is widely distributed in nature and presents a saprophytic mycelial form on plant debris and soil. In addition to humans, many different animal species, including cats, dogs, rabbits, can be infected by *S. schenckii* which can be isolated from domestic cats with or without sporotrichosis [3], [4]. Therefore, sporotrichosis is considered a zoonosis. People with occupational exposure to contaminated animals, plant materials and soil, such as farmers, florists, gardeners, are at high risk of infection [1], [2].

The traumatic inoculation of conidia and hyphae of *S. schenckii* results in the development of subcutaneous mycosis, in which the fungus differentiates into its yeast form. The subcutaneous infection is usually confined in the local skin, although it can spread along the lymphatics. Systemic sporotrichosis is rare and reported only to be associated with immunocompromised patients [5], [6]. Generally, cutaneous sporotrichosis is reported to be involved in activation of host Th2 response and cause granulomatous inflammation, including infiltration of lymphocytes, plasma cells, macrophages, epithelioid cells, and multinucleated giant cells [5].

In our previous experiment to characterize the clinical features of cutaneous sporotrichosis, we established a disease model in immunocompetent Wistar rats. However, most of the rats failed to manifest the typical granuloma in skin. Upon necropsy, many cream colored cysts were found in the hepatic parenchyma of these failed rats and the cysts were identified as *Cysticercus fasciolaris*, the larval form of *Taenia taeniaeformis*, according to the morphology described by Mahesh [7].

*T. taeniaeformis* is a cestode belonging to the family *Taenidae*, and it occurs as adult tapeworms in the small intestine of carnivorous definitive hosts and is transmitted through eggs in feces to rodents which serve as intermediate host in which the eggs develop into fluid-filled larvae in various organs, mainly in liver. In spite of frequent infections of *T. taeniaeformis* found in wild rats, it is rare in laboratory animals as only one has been reported in Wistar rats [7]. *T. taeniaeformis* belongs to parasitic helminthes, which have developed complex mechanisms to escape or modulate host immunity [8]. Helminths are known to induce immune anergy, anti-inﬂammatory responses [9], [8], which may be responsible for the failure of subcutaneous sporotrichosis model.

Helminth and fungus concomitant infection are common in developing countries. In China, cats are an important infection source of *S. schenckii* and *T. taeniaeformis.* We speculate that *S. schenckii* and *T. taeniaeformis* co-infection might take place in farmers. The influence of helminth infection on sporotrichosis remains less understood. In the present study, we developed an animal model for *T. taeniaeformis-S. schenckii* co-infection and single *S. schenckii* infectionand compared the pathology of cutaneous sporotrichosis, the expression of an important anti-fungal pattern cognition receptor dectin-1 and cytokine IL-17 and other Th1 and Th2 cytokines to gain insights into immunological mechanisms.

## Results

### Murine Cysticercosis Model

**Figure 1:**
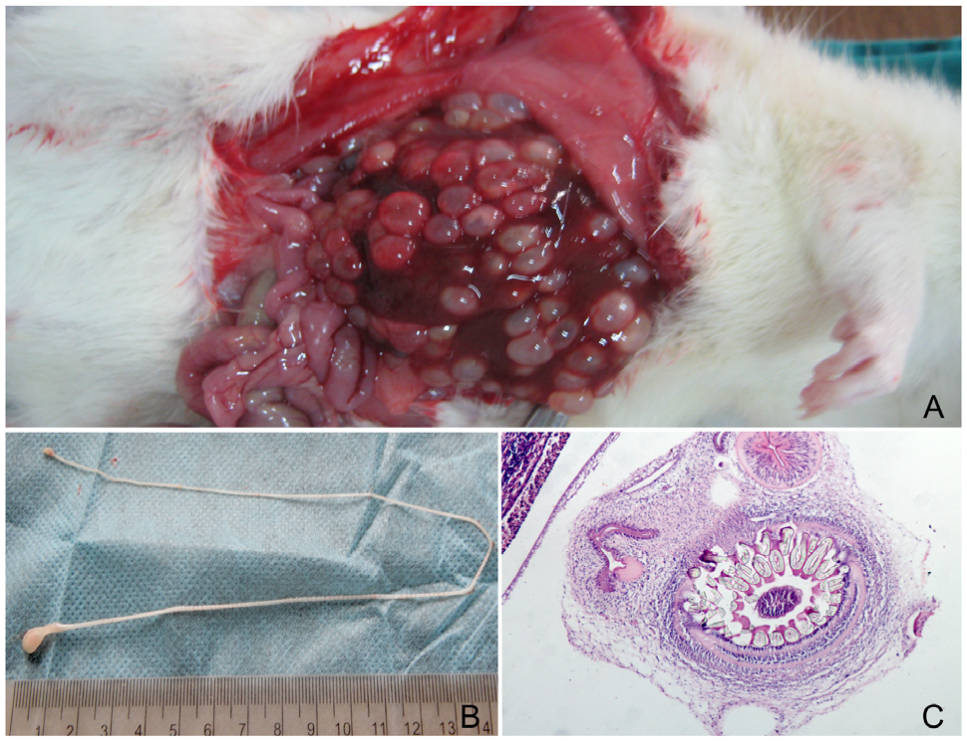
Morphology of *Taenia taeniaeformis*. (A) Taenia cyst covered the entire liver; (B) *Taenia taeniaeformis* larva with large scolex, long neck and pseudo segmentation of entire body length with terminal bulged portion. (C) Histology of a *Taenia* cyst revealed armed rostellum characterized by 2 rows of hooks and four suckers.

During necropsy of the 50 Wistar rats, cysts were found in the liver of all 20 rats in the co-infection group. No cyst was found in the liver of the rats in the other groups. Cysts in the liver appeared in most of the cases as milk-white in color, and were deeply attached to the caudal and lateral lobe of the liver (Fig. 1). The size of the cysts was 0.4–0.8 cm in diameter. Three rats each had five cysts and one rat had seven cysts present in the lateral and medial lobes of the liver. No clinical signs of diarrhea, weight loss, or lethargy were observed in co-infection group. Each cyst had a thick fibrous capsule surrounding the loosely coiled white-colored cestode larva in cream-colored thick ﬂuid. The viable larva was 15–30 cm in length and had a large scolex with a long neck (strobila, 3–4 cm) and pseudo segmentation of the entire body length and a terminal bulged portion. Stained parasites demonstrated typical taeniae features. The presence of an armed rostellum characterized by two rows of hooks and four suckers is the typical morphology of *T. taeniaeformis*.

### Fungus Infection

**Figure 2:**
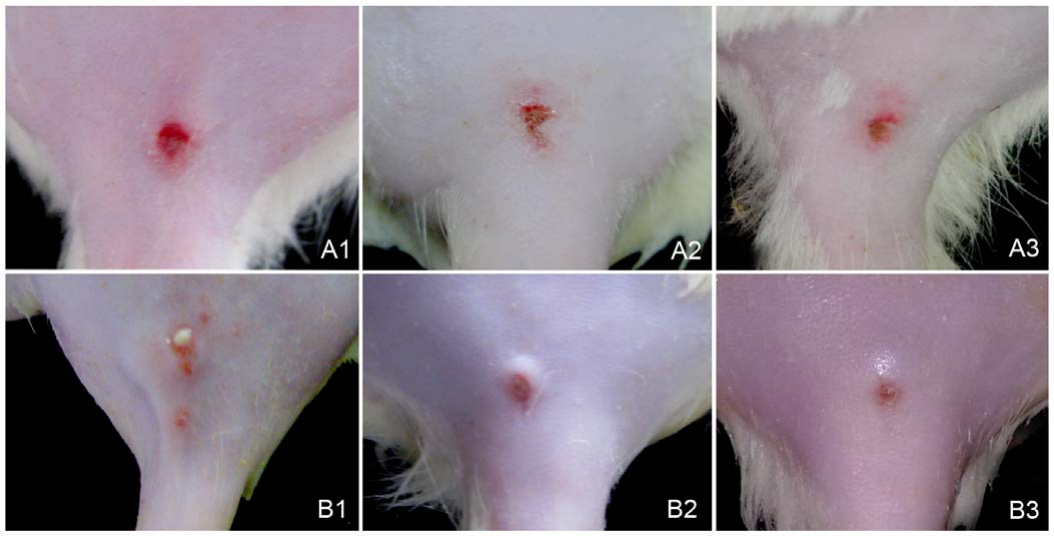
Lesions of the co-infected group (A) and fungus-infected group (B): 1–3 showed the 2^nd^, 3^rd^ and 5^th^ weeks post-inoculation (PI).

A subcutaneous soft lump at the injection site arose at the 2^nd^ to 3^rd^ day post-inoculation and their size was measured every day and the size reached a peak at the 5^th^ day in both infection groups (Fig. 2). These lesions slowly became nodules and reached their maximum size at the 1^st^ week post *S. schenckii* infection in the fungus-infected rats (with 0.90±0.08 cm in diameter) and at the 3rd week in co-infected rats (with 0.73±0.22 cm in diameter) respectively. After, these lesions in both groups began to shrink. The average size of the nodules in the co-infection group decreased to 0.38±0.23 cm in diameter at the 5^th^ week post *S. schenckii* inoculation. Meantime, nodules in the fungus-infection group decreased to 0.48±0.15 cm in diameter (Table 1). There was a significant difference in the size change of nodules between the co-infected and the fungus-infected groups (P = 0.002). In the co-infection group, the surfaces of the lesions underwent necrosis and developed into ulcers from the 5^th^ day and almost 80% of the nodules ulcerated at the 2^nd^ week post-inoculation. In the fungus-infection group, the same phenomenon was observed after the 10^th^ day and only 30% of the nodules were ulcerated at the 2^nd^ week. After ulceration, red-dark crusts were observed on the surface of the lesions, and these slowly became slight depressions with thin black crusts over the next 3 weeks in both groups. No lesions were observed at the inoculated site in the control group.

**Table 1:** Sizes of skin lesions in rat with co-infection, fungal-infection alone, and no infection (control group).

Time(wk)	Co-infection(n = 20)	Fungal-infection(n = 20)	Control group(n = 10)
1	0.68±0.27	0.90±0.08	0.00
2	0.65±0.23	0.78±0.15	0.00
3	0.73±0.22	0.58±0.18	0.00
4	0.53±0.28	0.50±0.18	0.00
5	0.38±0.23	0.48±0.15	0.00

### Fungal Culture

After culturing for 1 week at 27°C, moist, flat, folded surface colonies were found on Sabouraud dextrose agar incubated with aspirated pus or crust from bothco-infected and fungus-infected groups. The fungal culture for all the lung, liver, spleen, and kidney samples taken at different time intervals were negative under the same culture conditions. This indicated *S. schenckii* did not disseminate to the internal organs of the rats.

### Histopathology of the Internal Organs

Histopathological observation of liver sections from the co-infection group revealed abundant fibrous tissue around the cysticercus. Infiltration of eosinophilic granulocytes, lymphocytes, and plasma cells were also observed around the cysticercus in the liver sections. Lobular architecture was intact, but there were some cholestatic liver cells. No cysticercus was observed in the other systemic organs.

### Histopathology of the Skin Lesions

**Figure 3:**
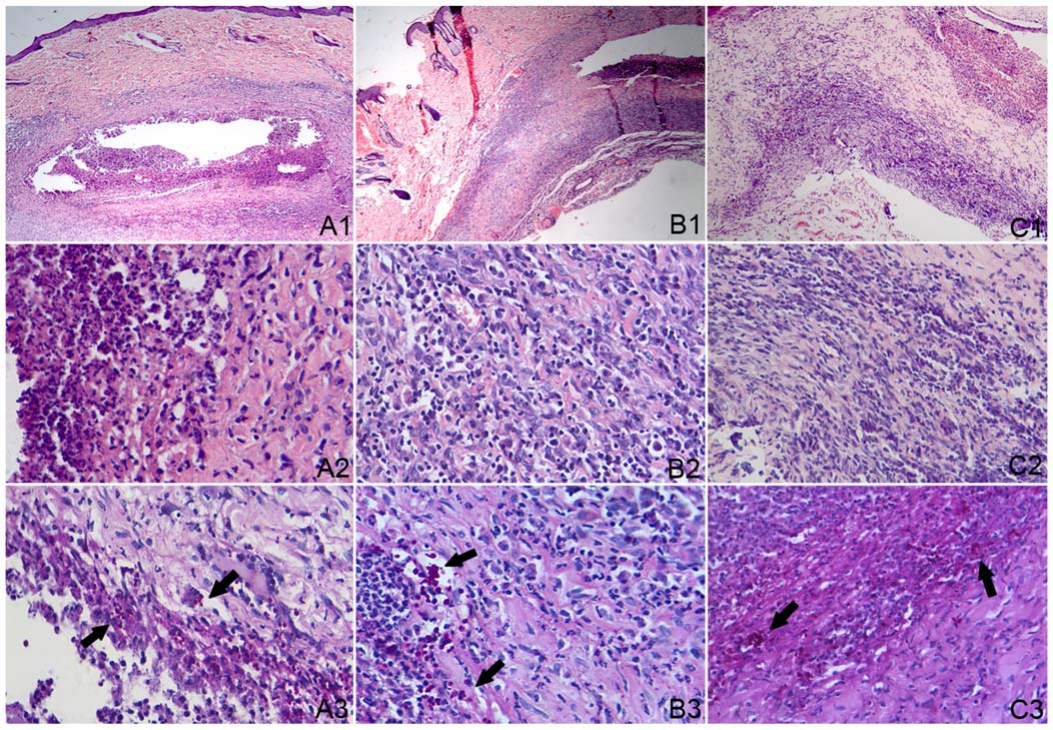
Histopathologic examinations by H&E (1 and 2) and PAS (3) in co-infection group. A1, B1 and C1 showed different lever of suppurative granulomatous inflammation in the dermis tissue at the 2^nd^, 3^rd^ and 5^th^ weeks(×100); A2, B2 and C2 showed most of the inflammatory cells were histocytes and epithelioid cells around necrosis center (×400); A3, B3 and C3 showed *S. schenckii* cells (arrow) at the 2^nd^, 3^rd^ and 5^th^ weeks (×400).

**Figure 4:**
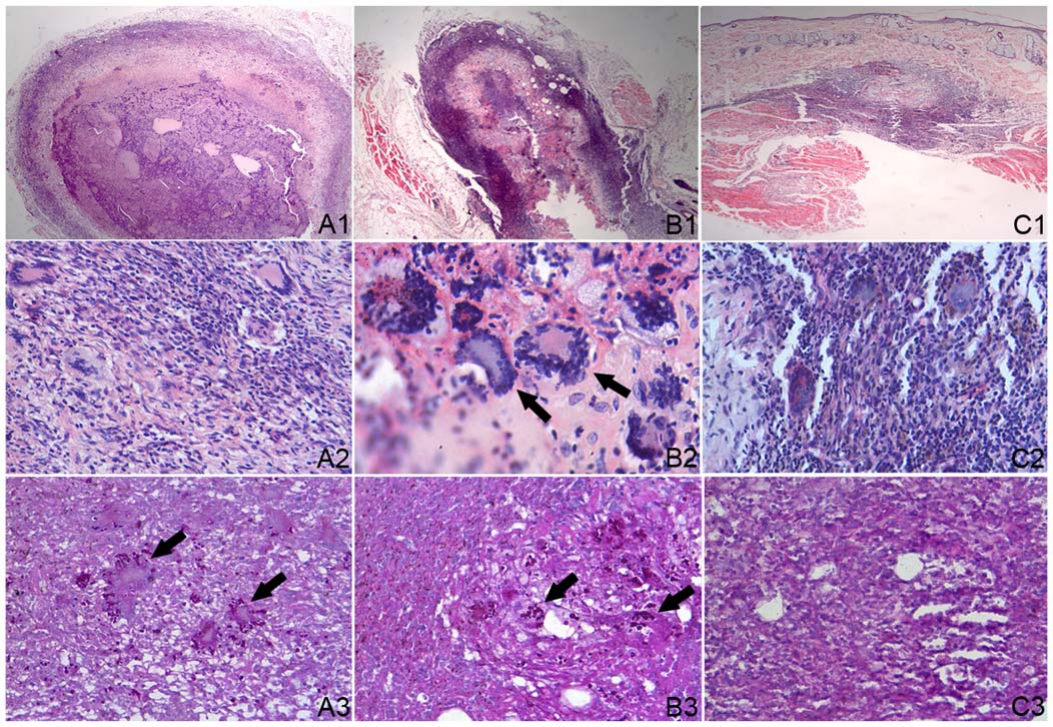
Histopathologic examinations by H&E (1 and 2) and PAS (3) in fungus-infection group. A1, B1 and C1 showed different level of suppurative granulomatous inflammation in the dermis tissue at the 2^nd^, 3^rd^ and 5^th^ weeks(×100); A2, B2 and C2 showed multinucleated giant cells (arrow) were formed except histocytes and epithelioid cells around necrosis center (×400); A3 and B3 showed *S. schenckii* cells (arrow) in multinucleated giant cells at the 2^nd^, 3^rd^, and no *S. schenckii* cell was observed in C3 at the 5^th^ weeks PI (×400).

**Figure 5:**
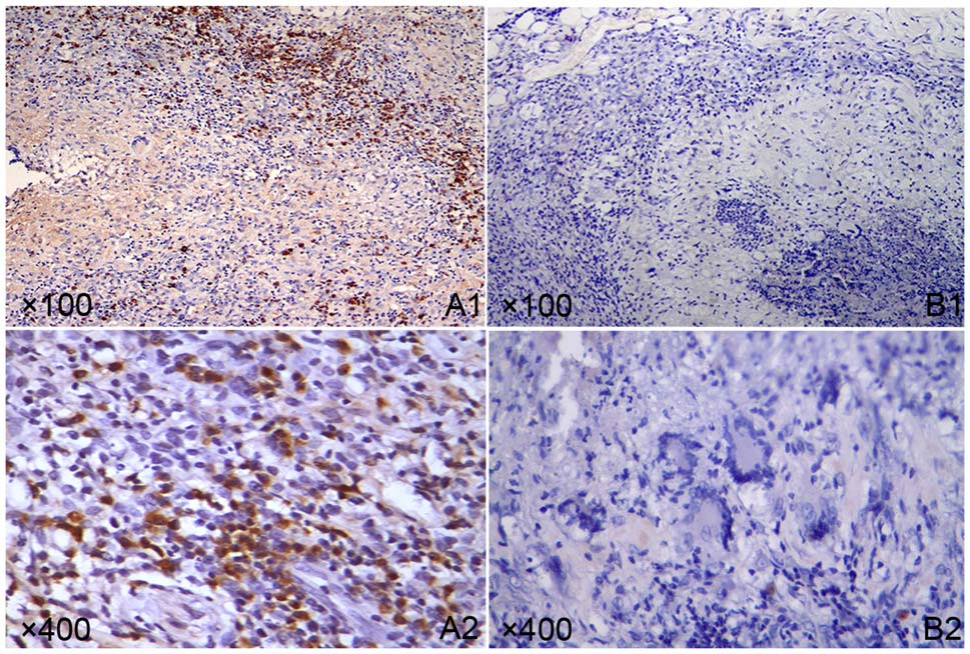
Dectin-1 immunohistochemical staining of skin lesions from the co-infected group (A) and fungus-infected group (B). A1–A2 showed a lot of positive cells in the lesion and mainly gathered in the tuberculosis-like layer. B1–B2 showed no positive cells.

The lesion in local skin infected with *S. schenckii* was tested by histological examination. At the 2^nd^, 3^rd^, and 5^th^ week post fungus-infection, the lesions in the co-infection and fungus-infection groups showed different levels of suppurative granulomatous inflammation following H&E staining, which was composed of extensive necrosis surrounded by an intense inflammatory band that consisted of epithelioid cells, multinucleated giant cells, neutrophils, the yeast forms of *S. schenckii*, and many lymphocytes in the outer layer of the lesions. An interesting finding was that there was no multinucleated giant cell in the lesions from the 2^nd^ to 5^th^ week in the co-infection group (Fig. 3), while in the fungus-infection group (Fig. 4) multinucleated giant cells were observed in the lesions at the 2^nd^ to 5^th^ week of infection. Moreover, the yeast forms of *S. schenckii* were observed in the multinucleated giant cells. On the other hand, at the 2^nd^ and 3^rd^ weeks of infection, many *S. schenckii* cells were observed in the nodules of both groups following Periodic acid-Schiff staining. At the 5^th^ week of infection in the co-infection group, many *S. schenckii* cells were still observed along with many neutrophils and mononuclear cells in the crustosus lesions, although the pus-like inﬂammatory focus was limited. In contrast, cell infiltrations, composed mainly of mononuclear cells, were observed in the crustosus lesions of the fungus-infection group, but *S. schenckii* cells were not found. The result of Dectin-1 immunohistochemical staining of the skin lesions is shown in Fig. 5. Positive cells were observed in the co-infected group and were mainly gathered in the tuberculosis-like layer. The number of positive cells reached a peak at the 3^rd^ week. In contrast, the Dectin-1–positive cells were not observed in the fungus-infected and control groups all the time. The histopathologic examinations of the inoculated sites of the control group demonstrated no abnormalities in the H&E and Periodic acid-Schiff staining.

### Cytokine Levels in Rat Serum

**Figure 6:**
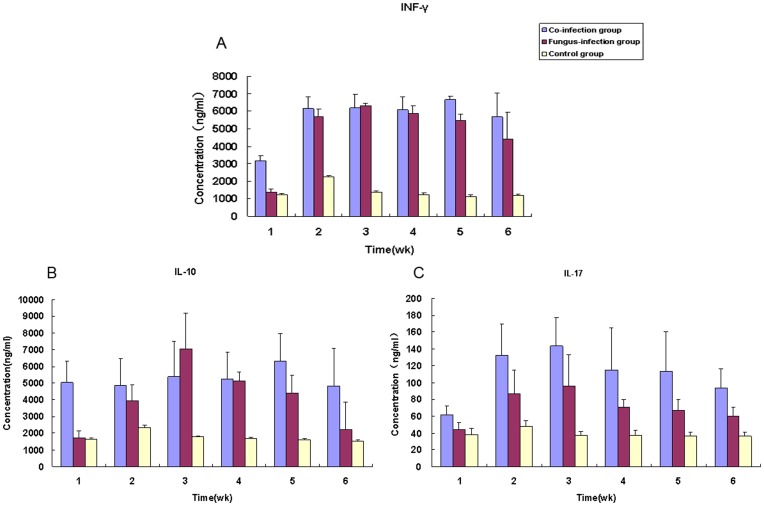
Cytokines IFN-γ, IL-10 and IL-17 released during *S. schenckii* infection and co-infection with *Taenia taeniaeformis* (A, B and C). IFN-γ and IL-10 (A and B) productions occurred in large amounts in the 1^st^, 2^nd^ and 3^rd^ weeks after *S. schenckii* infection in fungus-infected group, and reached the peak in the 2^nd^ week PI. IL-17 productions (C) of the co-infected group. There was a significant difference between the two groups for IL-17 levels (P<0.05).

Sporotrichosis is a granulomatous mycotic infection caused by *S. shenckii*. Granuloma formation, a critical event in the immune response against *S. shenckii*, is thought to be a result of the Th1 response in the host [10], [11]. Therefore, we determined whether the skin lesions of the co-infected group were more likely to ulcerate and whether the lack of multinucleated giant cell macrophages in the skin lesions from this group were due to an alteration in cellular responses associated with differences in Th1 and Th2 cytokine production. However, IL-12 and IL-4 were not detected in our experiment. IFN-γ production, as shown in Fig. 6A, kept increasing after *S. schenckii* infection, reached a peak in the 2^nd^ week in the fungus-infected group, and then decreased gradually. In the co-infection group, IFN-γ production was relatively high before *S. schenkii* infection and increased after *S. schenkii* infection and kept a stable high level until the 4^th^ week. IFN-γ production in the control group was low in all the observed time points. The same trends were observed in the production of IL-10 for the three groups (Fig. 6B). Except the pre-infection and the 4^th^ week post *S. schenckii* inoculation, no significant difference in serum IFN-γ was found between the co-infected and fungus-infected groups, and the same was true for IL-10 at the time points post *S. schenckii* infection.

Fig. 6C shows the concentration of IL-17 in the three groups. IL-17 production in the co-infected and the fungus-infected groups was the first to increase and reached a peak in the 2^nd^ week and then decreased. Moreover, IL-17 production in the co-infected group at every time point was higher than that of the fungus-infected group. There was a statistically significant difference between the two groups for IL-17 production (*P*<0.001).

## Discussion

Among parasites, helminths are potent inducers of immune anergy and anti-inﬂammatory responses as well as important agents that drive a Th2-biased immune response [8]. After *T. taeniaeformis* infection, wild-type BALB/c mice can develop a strong Th2-like response and produce high levels of IgG1, IgE, IL-5, and IL-4 [12]. However, in our experiments, the rats infected with *T. taeniaeformis* did not drive the Th2-bias response, as IL-4, the major Th2 cytokines couldn't be detected, while the Th1 cytokine IFN-γ was relatively high. This difference in immune response between mice and rats may be due to their genetic differences. On the other hand, IL-10, the major anti-inflammatory cytokine and the product of alternatively activated macrophages (M2), which characterizes helminthes infection, is largely produced. The profile of cytokines suggested that the induction of M2 was not driven by Th2 cytokines of acquired immune, but rather the innate immune directly by some products of the worm. Previous investigations suggested that *S. schenckii* of cutaneous origin can potently activate dendritic cells to induce a subsequently strong Th1-prone immune response, while those of visceral origin positively induce a Th2 environment as evidenced by significantly higher IL-4 production [13], [14]. The high level of IFN-γ in the two *S. schenckii* subcutaneous infection groups found in this study are in agreement with the results reported previously [10], [11]. However, IL-12, the cytokine priming Th1 response, was also undetectable in the two groups, and the productions of IFN-γ are all the result of activation of innate immune cells.

According to our results, the incidence rate of nodules in the co-infection group was equal to that of the fungus-infection group, but the average size of the nodules in the co-infection group was smaller than that of the fungus-infection group. Furthermore, the peak in nodules size changing was delayed 2 weeks, indicating the helminth infection attenuated inflammatory reaction against *S. schenckii*. Upon histological examination of the skin sections, a significant difference was observed between the co-infected and fungus-infected groups. In the co-infection group, there were many histocytes surrounding the necrosis lesion, but no multinucleated giant cells. In contrast, many multinucleated giant cells were observed in the fungus-infection group. In addition, at the 5th week of infection, many *S. schenckii* cells were still observed in the co-infection group, but were not in the fungus-infection group. It is well-known that in skin sporotrichosis, the histocytes in the lesions can phagocytize the fungal cells and fuse into a multinucleated giant cell and this is a very important anti-fungus infection mechanism [15]. In normal conditions, few free fungal cells are present in lesions. In our study, until to the 5^th^ week, free viable fungal cells can be found in the lesions of co-infected rats while they disappear in single fungal infection group. The defection of phagocytosis of fungus cells indicates that different innate immune responses exist between co-infection and fungal infection alone. According to the relatively high IL-10 level after *S. schenckii* infection, we speculate the alternatively activated macrophages (M2 cells) and the primary IL-10-producing innate cells are involved in anti-fungal infection. In rapid defense against pulmonary *Aspergillus fumigatus* infection, alveolar alternatively activated macrophages have a protective role through up-regulating the dectin-1, the specificβ-1,3 glucan receptor in the surface of dendritic cells, macrophages, and neutrophils that can bind to glucan exposed in fungal cells and mediate the phagocytosis of the fungal cells [16]. Dectin-1 expression was found to be highly up-regulated by GM-CSF and by the cytokines that induce alternative macrophage activation, IL-4 and IL-13, and down-regulated by IL-10, LPS, and dexamethasone, but not IFN-gamma [17]. Nevertheless, Modulation of dectin-1 receptor levels is independent of IL-4/IL-13 signaling [18]. Polysaccharides containing β-1, 3-glucoside, ether in insoluble particulate form or soluble form, such as zymosan or laminaran can up-regulate the expression of dectin-1 [19], [20]. In the excretory-secretory products (ESP) of helminth also contains β-1, 3-glucans which also induce expression of dectin-1 on the macrophages [21]. In this study, dectin-1 is highly expressed in the lesions of the rats of the co-infection group and is Th2 independent as IL-4 is undetectable. Its expression is probably induced by the ESP of cestode and the cyst liquid contains rich polysaccharides [22]. When binding to β-1, 3-glucans component of the fungal cell wall, dectin-1 is activated and mediates phagocytosis of the targeted fungus. The activated dectin-1 localizes to lipid raft microdomains for signaling and activating of phagocytosis and cytokine production in dendritic cells [23]. However, the dectin-1 expression in macrophages in the co-infection group failed in phagocytosis of *S. schenckii* cells. In addition, *S. schenckii* infection alone fails to induce expression of dectin-1, suggesting that *S. schenckii* lacks β-glicans and phagocytosis of *S. schenckii* is dectin-1 independent. The phagocytosis of *S. schenckii* cells is mediated by mannose receptor and complement receptor on the surface of phagocytes and is involved in CD4+ T cells and antibodies against *S. schenckii* [24], [25].

IL-17 is an important cytokine playing a role in anti-*Candida albicans* infection, however, in co-infected rats, the IL-17 expression is higher than that of fungal infected rats at every time points. The IL-17 increase doesn’t enhance the clearance of *S. schenckii*, suggesting that IL-17 doesn’t individually play a role in eliminating *S. schenckii*. Both individual infection of *T. taeniaeformis* and *S. schenckii* stimulate IL-17 production, but co-infection shows the additive effect. In the helminth infection, dectin-1 activated DC can convert Treg cells to Th17 [26], and IL-17 is induced by mannose receptor activated macrophage in response to *S. schenckii*, as well as *Candida albicans* [27]. Our study indicated that innate immunity based on dectin-1 and IL-17 is insufficient to eliminate *S. schenckii* infection, and CD4+ Th cells and antibody response are essential for clearance of *S. schenckii*. This may explain why cutaneous sporotrichosis mainly occurs in immunocompetent patients. Moreover, helminth infection protracts sporotrichosis. Thus, it is recommended that sporotrichosis patients should receive a parasite examination.

In conclusion, lack ofβ-glucans render *S. schenckii* unable to be eliminated by dectin-1-expressing phagocytes and IL-17 response, instead, acquired immune plays a predominant role in anti-*S. schenckii* infection.

## Materials and Methods

### Ethics Statement

This animal trial received approval by the Experimental Animals Ethics Committee of the Experimental Animal Center of Sun Yat-sen University and was finished in accordance with the ethics of animal experiments in the Experimental Animal Center of Sun Yat-sen University. Procedures involved fungus infection, collection of skin and blood samples and euthanasia. Fungus infection was performed by subcutaneous inoculation. Skin samples were taken by skin tissue biopsy surgery. Blood samples were taken from the tail vein. All invasive procedures were performed under anesthesia. The anesthetic method was the intra-peritoneal injection of 10% chloral hydrate. The anesthetic dose was 0.3 ml/100 g. At the end of the study, all animals were sacrificed by intra-peritoneal injection of excess 10% chloral hydrate. All efforts were made to minimize discomfort, pain and distress.

### Rodent Cysticercosis Model

This study used 50 female Wistar rats (4 weeks old) purchased from the laboratory animal center of Zhongshan School of Medicine, SYSU (Animal license number: SCXK Guangdong 2004-0011). Rats were assigned to one of three groups: the co-infection group and fungus-infection group of 20 rats each, and the control group of 10 rats. Before fungus infection, all rats were kept in sterile cages on wood-chip bedding and provided with a clean supply of water and commercial pelleted food for two months. In order to get the rats in the co-infection group naturally infected with *T. taeniaeformis*, totally about 10,000 *T. taeniaeformis* eggs were added to 100 gram food and mixed uniformly to make pellets equally assigned to each rats in co-infection group. The animals were inspected daily during the entire experiment.

### Fungus

The standard *S. schenckii* strain CMCC (F) D1a was provided by the Centre for Medical Microbiology Culture Collection of China (CMCC). This fungus shows dimorphism depending on the culture temperature; that is, the mycelial form occurs at 27°C and the yeast form occurs at 37°C. The organism was grown on Sabouraud dextrose agar (SDA) at 27°C for a week to obtain the mycelial form and then subcultured to potato dextrose agar (PDA) slants for seven days at 27°C to induce conidial formation. Conidial suspensions for in vitro infection were obtained from the strain by covering the fungal colony with 5 ml of sterile saline and gently rubbing the colonies with the tip of a transfer pipette. The resulting conidial suspensions were transferred to sterile 10-ml centrifuge tubes. Collected samples were filtered using sterile gauze, counted using a hemocytometer, and then adjusted to a suspension of a 1.0×10^8^ conidia/ml by adding sterile saline [28].

### Fungal Infection Experiments

A total of 100 µl of the *S. schenckii* conidia suspension was inoculated subcutaneously at the back (near the root of tail) of the rats in the co-infection and fungus-infection groups at 8th week after eggs exposure. The rats in the control group were inoculated subcutaneously with the same volume of 0.85% sterile saline at the same site. Rats were examined once a day for the first 14 days and at intervals of every 2 days for up to 5 weeks. The lesions and their sizes, duration, and tissue at the inoculation sites were observed. Direct smear of fungal culture were prepared from aspirated pus at the 2^nd^, 3^rd^, and 5^th^ week after inoculation. Furthermore, at the end of the studies, samples from the lungs, livers, and spleens of all rats were removed under aseptic conditions for fungal culture.

### Histological Analysis

Biopsy of the skin lesions was performed at the 2^nd^, 3^rd^, and 5^th^ week after *S. schenckii* inoculation. The skin samples were fixed in 10% formalin overnight for sectioning (in order to be embedded in paraffin according to procedures used for routine histology) and then stained with hematoxylin and eosin (H&E) and Periodic acid-Schiff. Livers with parasites were also kept in 10% formalin for H&E staining. For immunostaining of alternatively activated macrophages, the skin sections were stained using an anti-Dectin-1/CLECSF12 kit (diluted 1∶400; Bios, China) according to the manufacturer's recommendations.

### Detection of IL-12, IFN-γ, IL-4, IL-10, and IL-17 Levels in Rat Serum

Peripheral blood was collected every week from tail snips of all experimental rats. The blood was centrifuged to isolate serum and stored at −40°C until use. Th2-associated cytokines IL-4 and IL-10, Th1-associated IL-12 and IFN-γ, and Th17-associated IL-17 were measured using the ELISA method according to the manufacturer’s instructions (Cusabio, USA). The absorbance was read at 450 nm on a Titertek Multiscan MK3 microplate reader (Labsystems, Dragon, Finland), and cytokine concentrations were calculated using a curve of known concentrations of IL-12, IFN-γ, IL-4, IL-10, and IL-17 standards using Curve Expert1.3 software. The results were expressed in ng/ml.

### Statistical Analysis

Quantitative variables with a normal distribution were submitted to analysis of variance in repeated measurements and paired samples test using SPSS v16.0 (SPSS, Chicago, IL, USA), with a focus on comparing the co-infection versus fungal-infection alone. Statistical significance was accepted at *P*<0.05 in 2-sided tests.
